# Reported reasons for breakdown of marriage and cohabitation in Britain: Findings from the third National Survey of Sexual Attitudes and Lifestyles (Natsal-3)

**DOI:** 10.1371/journal.pone.0174129

**Published:** 2017-03-23

**Authors:** Kirsten Gravningen, Kirstin R. Mitchell, Kaye Wellings, Anne M. Johnson, Rebecca Geary, Kyle G. Jones, Soazig Clifton, Bob Erens, Michelle Lu, Chenchit Chayachinda, Nigel Field, Pam Sonnenberg, Catherine H. Mercer

**Affiliations:** 1 Department of Microbiology and Infection Control, University Hospital of North Norway, Tromsø, Norway; 2 Research Department of Infection & Population Health, University College London, London, United Kingdom; 3 MRC/CSO Social and Public Health Sciences Unit, Institute of Health and Wellbeing, University of Glasgow, Glasgow, Scotland; 4 Department of Social and Environmental Health Research, London School of Hygiene & Tropical Medicine, London, United Kingdom; 5 Department of Health Services Research & Policy, London School of Hygiene & Tropical Medicine, London, United Kingdom; 6 Department of Obstetrics and Gynaecology, Faculty of Medicine Siriraj Hospital, Mahidol University, Bangkok, Thailand; Birmingham City University, UNITED KINGDOM

## Abstract

**Objectives:**

Breakdown of marriage and cohabitation is common in Western countries and is costly for individuals and society. Most research on reasons for breakdown has focused on marriages ending in divorce and/or have used data unrepresentative of the population. We present prevalence estimates of, and differences in, reported reasons for recent breakdown of marriages and cohabitations in Britain.

**Methods:**

Descriptive analyses of data from Britain’s third National Survey of Sexual Attitudes and Lifestyles (Natsal-3), a probability sample survey (15,162 people aged 16–74 years) undertaken 2010–2012, using computer-assisted personal interviewing. We examined participants’ reported reasons for live-in partnership breakdown in the past 5 years and how these varied by gender and partnership type (married vs. cohabitation).

**Results:**

Overall, 10.9% (95% CI: 9.9–11.9%) of men and 14.1% (13.2–15.0%) of women reported live-in partnership breakdown in the past 5 years. Mean duration of men’s marriages was 14.2 years (95% CI: 12.8–15.7) vs. cohabitations; 3.5 years (3.0–4.0), and for women: 14.6 years (13.5–15.8) vs. 4.2 years (3.7–4.8). Among 706 men and 1254 women reporting experience of recent breakdown, the reasons ‘grew apart’ (men 39%, women 36%), ‘arguments’ (27%, 30%), ‘unfaithfulness/adultery’ (18%, 24%, p<0.05), and ‘lack of respect/appreciation’ (17%, 25%, p<0.05) were the most common, irrespective of partnership type. A total of 16% of women vs. 4% of men cited domestic violence. After adjusting for age at interview and duration of partnership, there were no significant differences in reasons given for breakup by partnership type, except that men more commonly cited ‘moving due to changing circumstances’ as a reason for a cohabitation ending than for a marriage (AOR = 3.78, 95% CI: 1.08–13.21); and among women, ‘not sharing housework’ (0.54, 0.35–0.83) and ‘sexual difficulties’ (0.45, 0.25–0.84) were less commonly cited as reasons for cohabitation ending than marriage.

**Conclusion:**

These representative data on recently ended marriages and cohabitations among men and women in Britain show that there were more similarities than differences in the reasons reported for breakdown across partnership type. For both marriages and cohabitations, cited reasons relating to communication and relationship quality issues were most common, followed by unfaithfulness/adultery. Our findings support a focus on relationship quality, including communication and conflict resolution, in preventive and therapeutic interventions addressing breakdown of live-in partnerships.

## Introduction

Live-in partnership breakdown, whether that of a marriage or a cohabitation, is relatively common in contemporary Western countries [1, 2] and causes significant distress, hence the study of dissolution of partnerships is of interest to policy-makers and society more broadly. A number of studies have sought to identify sociodemographic and interpersonal factors associated with partnership breakdown [3–7], but few studies have looked at the reasons people report for breakdown, and those that have tend to be conducted in unrepresentative samples and/or have focused on marriages ending in divorce [8–11]. The need for studies addressing not only breakdown of marriage but that of cohabitation in the general population over a broad age range has also been identified [12].

In Britain and Europe, marriage rates have been steadily declining in recent decades, reducing the proportion of the population at risk of divorce [13], while more couples now cohabit and for longer [1, 14, 15]. Since the early 1990s, the majority of couples choose to live together before marriage, but fewer cohabiting couples now end up marrying and more of them separate without ever getting married [14]. Although the UK Office for National Statistics compiles statistics on divorces, reasons are limited to broad categories restricted by legal definitions and may not reflect individuals’ own perceptions [1]. Data on the formation and dissolution of cohabiting partnerships are not routinely collected in Britain and, consequently, there are few studies on the breakdown of cohabitations despite these being the fastest growing form of live-in partnerships [1]. However, long-term partnership stability is more common among married than among cohabiting couples [4, 12].

Differences in the reasons cited for divorce have been observed between countries [5] over time, reflecting cultural and historical differences in social attitudes towards gender roles and marriage, and in legislation facilitating divorce [9, 16, 17]. A Dutch national survey of different divorce cohorts from 1949 to 1996 observed a trend towards reporting reasons such as growing apart, not getting enough attention, and problems related to managing work and household duties, particularly among women, while infidelity and violence as reported reasons declined in prevalence over time [9]. Domestic violence, which has been shown to have deleterious effects on physical and emotional health, remains a significant problem in Britain [18] and is frequently reported as a reason for divorce by women but only rarely by men [8, 9, 11, 19, 20]. Several studies have shown that women are more likely than men to specify a larger number of reasons [8, 21] and to provide more complex explanations for live-in partnership breakdown [11] and that women more often than men report motives such as infidelity, unhappiness and money problems [4, 8, 11, 19].

Live-in partnership breakdown is costly both emotionally and financially with the cost to UK taxpayers estimated at £47 billion in 2015 in legal aid, lost work hours, housing support and other related factors [22, 23]. The evidence is that divorce has a negative impact on the well-being and physical and mental health of adults and children [5, 24]. Up-to-date knowledge of the reported reasons for dissolution of marriage and cohabitation at a population level may also be important for relationship counsellors, for lawyers working in the area of probate, and for men and women attempting to evaluate their own experience. Furthermore, this knowledge may be useful for guiding preventive interventions by informing relationship and marriage advice. We used data from Britain’s third National Survey of Sexual Attitudes and Lifestyles (Natsal-3), a representative sample of the population, to present prevalence estimates of, and differences in, reported reasons for recent breakdown of marriages and cohabitations among men and women in Britain.

## Materials and methods

Full details of the methods used in Natsal-3 have been reported elsewhere (34, 35). Briefly, we used a multistage, clustered, stratified probability sample design. A total of 15,162 men and women aged 16–74 years (6,293 men) living in private households in Britain, were interviewed between September 2010 and August 2012. The response rate was 57.7% (of all addresses known or estimated to be eligible) which is consistent with other population-based surveys completed around the same time, and the co-operation rate was 65.8% (of all addresses known to be eligible) [25, 26].

Participants were interviewed using a combination of face-to-face computer-assisted personal interview (CAPI), followed by computer-assisted self-interview (CASI), and then a final CAPI. Participants who reported the breakdown of a live-in partnership (of at least one month duration) in the 5 years prior to the interview, were asked in the second CAPI why the partnership ended by showing them a card that listed 12 predefined reasons, such that participants only had to report a letter code to the interviewer (Table 1).

**Table 1:** Questions used to assess reasons for live-in partnership breakdown.

Topic	Eligible respondents	Question wording	Responses allowed	Reasons displayed in random order on showcards
Reported reasons for breakdown of most recent live-in partnership past 5 years	1,960 sexually-experienced respondents (706 men and 1254 women) reporting breakdown of most recent live-in partnership the past 5 years prior to the interview	Why did your relationship with this partner end–can you just tell me the code letters?	Multiple	D) Death of partner (if yes, not asked further questions)
				E) Difficulties with sex life
				F) Other (specify at next question)*
				J) Domestic violence
				K) Different interests/nothing in common
				N) Unfaithfulness/adultery
				Q) Arguments
				R) Not having children
				S) Grew apart
				V) Moved because of change in circumstances (e.g. changed job)
				X) Lack of respect or appreciation
				Y) Not sharing enough housework
				Z) Money problems
Specification of ‘other’ reason for live-in partnership breakdown	110 sexually-experienced respondents (38 men and 72 women) reporting ‘other’ as reason for breakdown of live-in partnership the past 5 years prior to the interview	If F) *Other, please type in the specific reason(s)	Free text	*Specific reasons typed in by respondents
				1) Drinks/drugs/gambling problems
				2) Mental health or related problems
				3) Another relationship involved
				4) Problems with children
				5) Never at home
				6) Problems with parents/in-laws/family
				7) Partner left without explanation
				8) Age problem
				9) Lived in/moved to a different country
				10) Changed mind/feelings/personality

Multiple reasons could be reported. Participants who had previously stated their marital status as “widowed”, or who reported “death of partner” as the reason for relationship breakdown, were not asked any further reasons, and were therefore excluded from the analysis. Here, we present primarily descriptive data for sexually-experienced (i.e. those reporting one or more sexual partners ever) men and women aged 16–74 years who reported the breakdown of a live-in partnership (either opposite-sex or same-sex partnership) that they had been in during the 5 years prior to interview and at least one reason why this partnership ended. We present prevalence estimates of, and differences in, reported reasons for breakdown of the most recent ended live-in partnership, by gender and partnership type.

### Statistical analyses

**Fig 1:**
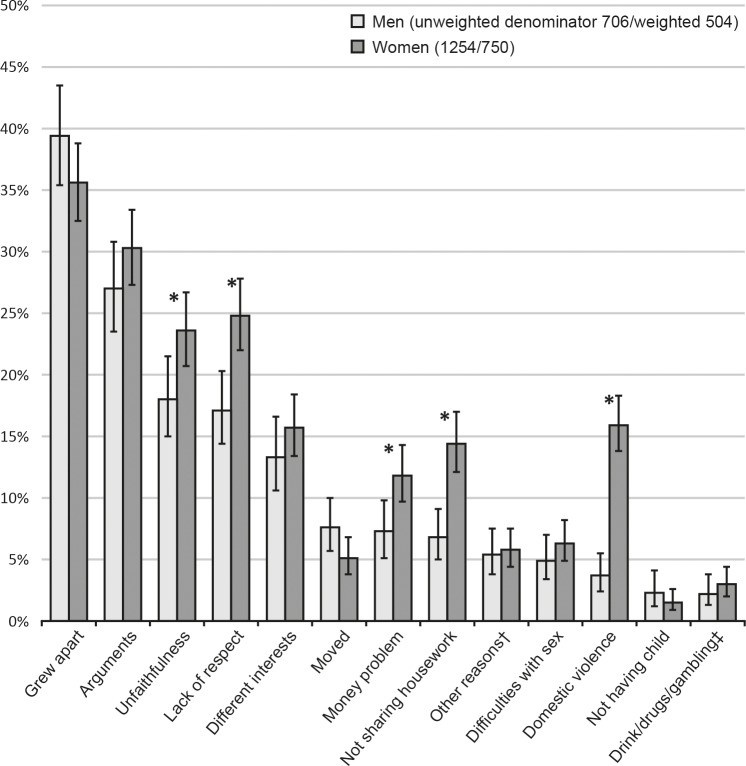
Reasons for live-in partnership breakdown reported by men and women aged 16–74 in Britain (presented in order of men’s prevalence). *; p-value <0.05 for gender difference (Chi-square test). †; those answering ‘other reasons’ were asked to specify their reason. ǂ; drink/drugs/gambling was the only specified reason reported by >1% of respondents. Each bar includes 95% confidence intervals.

We did all analyses using the complex survey functions of STATA (v13) to account for the stratification, clustering, and weighting of the Natsal-3 data [26]. We present primarily descriptive statistics by gender and previous live-in partnership type (married *vs*. cohabitated) to examine how the reported reasons for live-in partnership breakdown vary. Weighted prevalence estimates, means, and 95% confidence intervals (CIs) are presented. The Chi-square test was used to calculate *p*-values for the difference between proportions (Fig 1). To analyse how the reported reasons for the ended partnership (independent variable) vary by partnership type pre-breakdown (outcome variable), logistic regression was used to calculate odds ratios adjusted for participants’ age at interview and the duration of the recently ended partnership (AOR) for each reason. A Wald test was used to calculate global *p*-values for the logistic regression analyses. Proportional Venn diagrams were used to present the overlap between the most common reasons by gender and by most recent ended partnership type.

### Ethical approval

All Natsal-3 participants were given an information leaflet to read prior to participating in the survey and had the opportunity to discuss with the interviewer. Verbal informed consent was obtained for participation in the interview and interviewers had to confirm that participants had read the information leaflet before commencing the interview. In line with standard practice for UK surveys, and in response to evidence suggesting that signing a consent form might lead to a greater sense of obligation to complete the interview, we obtained verbal rather than written consent. The Natsal-3 study, including the consent procedures, was approved by the Oxfordshire Research Ethics Committee A (Ref: 09/H0604/27). All participants provided their own consent to participate, however, for 16–17 year-olds living at home, a parent/guardian provided additional verbal assent for participation.

## Results

### Prevalence of recent ended partnerships

Overall, 10.9% (95%CI: 9.9–11.9%, weighted percentage; unweighted n = 825) of men and 14.1% (95%CI: 13.2–15.0; n = 1467) of women in Natsal-3 reported the end of a live-in partnership (including 70 men and 144 women who cited the death of a partner) in the 5 years prior to interview. Of these, 706 men and 1254 women reported at least one reason for why their most recent partnership ended, excluding death of partner, which is the population of interest hereon. Among this population, 138 men and 224 women reported more than one ended live-in partnership in the 5 years prior to interview; data is presented for their most recent breakdown only.

### Sociodemographic and partnership characteristics

Men who were married: mean age at interview 46.4 (95% CI: 44.8–47.9). Men who cohabited: mean age at interview 33.8 (95% CI: 32.7–34.9). Mean age at interview for men who had been married was 46.4 years (95%CI: 44.8–47.9) and 33.8 years (32.7–34.9) for men who had cohabited. The corresponding means for women were 43.5 years (42.3–44.7) and 31.8 years (31.0–32.6), respectively. Looking at the characteristics of the most recent ended partnerships, the mean age at the start of living together for men was similar whether they had been married at the start: 29.7 years (28.4–31.0) or cohabited: 28.5 years (27.5–29.4), and similarly for women: 26.9 years (25.9–28.0) and 25.9 years (25.3–26.5), respectively. About one in six men and women were married from the start of living together, one in six cohabited then married, and two-thirds only ever cohabited (data not shown). Mean duration of men’s ended marriages was 14.2 years (12.8–15.7), while for cohabitations it was 3.5 years (3.0–4.0). Corresponding numbers for women were 14.6 years (13.5–15.8) and 4.2 years (3.7–4.8), respectively. Altogether, 1.9% (1.0–3.5) of men’s and 3.5% (2.1–5.9) of women’s ended live-in partnerships had been with a same-sex partner.

### Reported reasons for breakdown

The most commonly reported reasons reported for live-in partnership breakdown by both men and women were that they grew apart, followed by arguments, unfaithfulness/adultery, and lack of respect/appreciation (Fig 1).

A similar proportion of men and women reported having grown apart (39.4% and 35.6%, respectively), arguments (27.0% and 30.3%), different interests/nothing in common (13.3% and 15.7%), other reasons (5.4% and 5.8%), difficulties with sex (4.9% and 6.3%), not having children (2.3% and 1.5%), and drink/drugs/gambling (2.2% and 3.0%). More women than men reported unfaithfulness (23.6% vs. 18.0%), lack of respect/appreciation (24.8% vs. 17.1%), money problems (11.8% vs. 7.3%), and not sharing housework (14.4% vs. 6.8%). Domestic violence was reported four times as often by women as by men (15.9% vs. 3.7%). The remaining reasons were reported by less than 1.0% of participants (Table 1).

About half the participants gave only one reason for breakdown, one in five gave two, and a little over one in four gave three or more reasons; in total an average of just under two reasons per participant (data not shown). More men than women (56.3% vs. 48.8%) reported a single reason, one-fifth of both men and women (19.5% and 20.3%) reported two, and less men than women reported three or more (24.2% vs. 30.9%).

**Fig 2:**
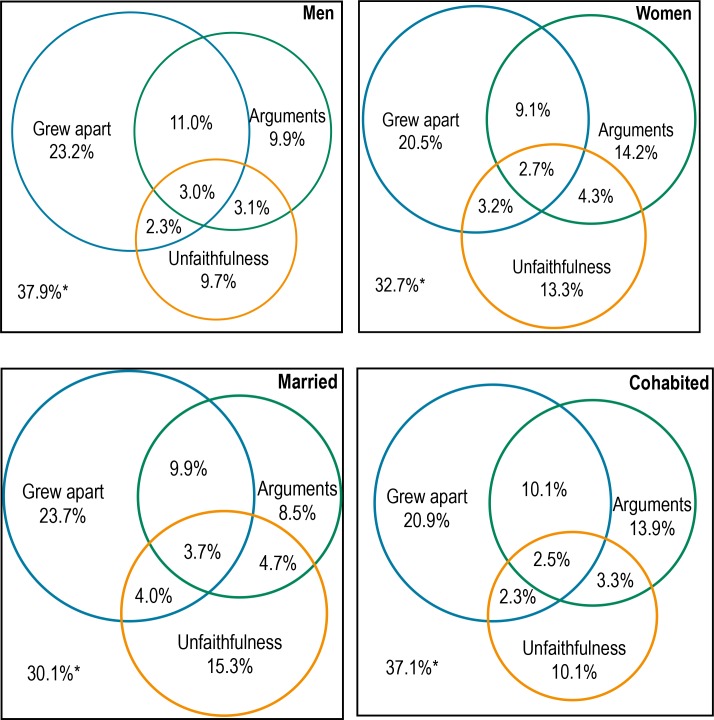
Venn diagrams of the most common reasons for live-in partnership breakdown by gender and most recent ended partnership type. (A) Men. (B) Women. (C) Formerly married men and women. (D) Formerly cohabiting men and women. *37.9% of men, 32.7% of women, 30.1% of formerly married men and women, and 37.1% of those who cohabited reported none of these reasons.

Roughly two-thirds of both men and women cited one or more of the three most prevalent reasons: grew apart, arguments, and unfaithfulness/adultery (Fig 2A and 2B).

Participants reporting arguments or unfaithfulness/adultery were equally likely to give the other common reasons but this was not the case for grew apart which had less overlap. The patterns of the overlapping areas were generally similar between genders.

Looking at the reported reasons for breakdown by partnership type, there were more similarities than differences (Tables 2 and 3).

**Table 2:** Reported reasons for live-in partnership breakdown among men in Britain aged 16–74, by most recent ended partnership type.

	Men							
	Married		Cohabited					
*Unweighted*, *weighted denominators*	*182*, *191*		*520*, *459*					*Denominat*.
	%	(95% CI)	%	(95% CI)	AOR	(95% CI)	p-value	*Unw*.,*weigh*.
Grew apart	41.7	(34.2–49.6)	38.5	(33.8–43.3)	1.43	(0.85–2.41)	0.175	*290*, *256*
Arguments	26.1	(19.9–33.5)	27.5	(23.4–32.1)	1.20	(0.70–2.07)	0.514	*206*, *176*
Unfaithfulness/adultery	24.5	(18.4–31.8)	15.5	(12.7–19.3)	0.77	(0.43–1.39)	0.382	*130*, *117*
Lack of respect/appreciation	22.0	(16.3–29.0)	15.2	(12.2–18.9)	0.83	(0.45–1.51)	0.535	*126*, *112*
Different interests/nothing in common	17.6	(12.3–24.6)	11.6	(8.7–15.2)	1.00	(0.51–1.96)	0.996	*91*, *87*
Move because of change in circumstances	2.2	(1.0–5.0)	9.9	(7.4–13.2)	3.78	(1.08–13.21)	0.037	*59*, *50*
Money problems	11.0	(6.7–17.4)	5.8	(3.9–8.5)	0.68	(0.31–1.48)	0.329	*53*, *48*
Not sharing enough housework	8.1	(4.9–13.0)	6.3	(4.3–9.2)	0.91	(0.38–2.19)	0.839	*51*, *44*
Other reasons	7.2	(4.2–12.1)	4.7	(3.0–7.2)	0.77	(0.32–1.84)	0.562	*39*, *35*
Difficulty with sex	9.4	(5.5–15.5)	3.0	(1.8–5.0)	0.57	(0.20–1.62)	0.293	*35*, *32*
Domestic violence	5.7	(3.0–10.7)	2.8	(1.7–4.8)	N/A		N/A	*27*, *24*
Not having child	1.7	(0.5–5.3)	2.5	(1.3–5.0)	N/A		N/A	*14*, *15*

Notes for Table 2: CI, confidence interval; AOR, odds ratio adjusted for age at interview and duration of the most recent ended live-in partnership (reference category: previously married); *p-*value, global *p-*value calculated using a Wald test (for each reason); N/A, not applicable due to small numbers.

**Table 3:** Reported reasons for live-in partnership breakdown among women in Britain aged 16–74, by most recent ended partnership type.

	Women							
	Married		Cohabited					
*Unweighted*, *weighted denominators*	*343*, *243*		*828*, *440*					*Denominat*.
	%	(95% CI)	%	(95% CI)	AOR	(95% CI)	p-value	*Unw*.,*weigh*.
Grew apart	41.0	(34.9–47.4)	33.2	(29.7–36.9)	1.09	(0.75–1.60)	0.639	*437*, *267*
Arguments	27.4	(22.0–33.6)	31.7	(28.4–35.3)	1.07	(0.73–1.56)	0.736	*402*, *227*
Unfaithfulness/adultery	30.2	(24.5–36.6)	20.5	(17.6–23.6)	0.74	(0.51–1.07)	0.110	*293*, *177*
Lack of respect/appreciation	32.4	(26.7–38.7)	21.2	(18.3–24.5)	0.70	(0.48–1.02)	0.062	*301*, *186*
Different interests/nothing in common	20.5	(15.5–26.6)	13.5	(11.1–16.3)	0.83	(0.53–1.30)	0.416	*175*, *118*
Move because of change in circumstances	2.4	(0.8–7.0)	6.5	(4.9–8.5)	1.19	(0.31–4.57)	0.795	*67*, *38*
Money problems	16.3	(11.9–21.8)	9.8	(7.7–12.3)	0.63	(0.39–1.01)	0.055	*145*, *89*
Not sharing enough housework	21.2	(16.2–27.2)	11.2	(9.1–13.7)	0.54	(0.35–0.83)	0.005	*176*, *108*
Other reasons	6.8	(4.2–10.9)	5.3	(3.8–7.3)	0.96	(0.49–1.85)	0.892	*67*, *43*
Difficulty with sex	11.6	(8.1–16.5)	3.8	(2.7–5.4)	0.45	(0.25–0.84)	0.011	*71*, *47*
Domestic violence	16.0	(12.1–20.8)	15.5	(13.0–18.2)	0.74	(0.48–1.16)	0.188	*214*, *117*
Not having child	0.9	(0.3–2.5)	1.9	(1.0–3.4)	N/A		N/A	*20*, *12*

Notes for Table 3: CI, confidence interval; AOR, odds ratio adjusted for age at interview and duration of the most recent ended live-in partnership (reference category: previously married); *p-*value, global *p-*value calculated using a Wald test (for each reason); N/A, not applicable due to small numbers

In analyses adjusted for age at interview and duration of the most recent ended live-in partnership, men who cohabited were more likely than those who were married to cite the reason moving because of change in circumstances (AOR 3.78) (Table 2). Women who cohabited were less likely than those who were married to cite not sharing housework (AOR 0.54) and difficulty with sex (AOR 0.45) (Table 3) as reasons for their partnership ending. Venn diagrams for participants who were married versus cohabited showed a similar pattern of overlap between the three most commonly cited reasons (Fig 2C and 2D).

## Discussion

### Statement of main findings

These nationally representative data on recently ended live-in partnerships in Britain confirm that marriages are of significantly longer duration than cohabitations. In spite of this, we detected more similarities than differences in the reasons cited for breakdown of marriages and cohabitations after adjusting for age at interview and duration of partnership. Approximately half of participants reported multiple reasons for breakdown with overlap between the most common reasons, which were ‘grew apart’, ‘arguments’ and ‘unfaithfulness/adultery’, independent of partnership type. One in six women cited domestic violence while very few men gave this as their reason for their partnership breakdown. Difficulty with sex was reported by one in 20 men and one in 16 women.

### Strengths & weaknesses of the study

A strength of this study is that it is based on a large probability-sample survey so that the data can be considered as broadly representative of the British population. To our knowledge, Natsal is the only large-scale representative study of men and women to provide data on the reported reasons for *recent* breakdown of marriage or cohabitation. Given the limited age range of previous Natsal studies, this paper provides the first data across a broad age range, corresponding to much of adulthood. Although Natsal-3 gave participants the option of reporting a number of reasons for the recent breakdown, the use of predefined categories limited the possibility of obtaining explanations for live-in partnership breakdown formulated by participants themselves. Reasons such as ‘grew apart’ and ‘arguments’ are broad categories, and likely reflect a complexity that is difficult to capture in the context of a broad survey such as Natsal. It is a limitation that participants in Natsal-3 were not asked to rank the reported reasons in order of importance. Further, as data were provided by one partner only it was not possible to compare the reasons individuals gave for partnership breakdown with those of their former partner. Nor were the questions able to establish the extent to which the conduct of each partner was implicated in the reasons for breakdown. There is research that suggests that individuals initiating the divorce may report different reasons from non-initiators [27] but Natsal-3 did not collect data on this. The evidence is also that reasons cited for divorce, and potentially partnership breakdown more generally, may change over time as an adjustment to the event [8, 9, 27]. However, as a cross-sectional study Natsal-3 was only able to capture the reasons participants reported at the time of the interview in contrast to longitudinal studies that can describe change over time. The reason(s) people gave for their partnership breakdown are subjective accounts in retrospect and may reflect the justifications that individuals make to themselves, as well as their sense of what is socially acceptable in their social context [9]. However, research can only ever capture what people report (*vs*. what they actually think or do). With these limitations in mind, we consider our data to be of high quality, from a survey with low item non-response as compared to social surveys undertaken contemporaneously, reflecting considerable resource put in to Natsal-3 in order to provide an environment that encourages participants to report sensitive data as close as possible to what they actually think and do [26].

### Our findings in relation to other studies

The longer duration of marriages compared with cohabitations suggests that they are different types of partnerships, possibly with different level of commitment, and confirm that cohabitation may not be a long-term arrangement for many couples in Britain [12, 15]. Nevertheless, more than two-thirds of couples who began cohabitating in 2000–2004 in Britain were either still cohabiting or had married 5 years later [14]. Different types of cohabiting partnerships with different levels of commitment and failure rates are described in the literature, such as trial marriage (testing ground for later marriage), a marriage-like partnership (indifferent to marrying), an alternative to marriage (a decision not to marry), or as an alternative to being single (living together while dating) [28], and participants in Natsal-3 who had experienced the end of a cohabitation were not asked to categorise their former partnership according to such criteria. The ended cohabitations are likely therefore to correspond to a mix of the different types of partnership.

Compared to analyses of similar data from Natsal-2 conducted a decade earlier, we chose not to aggregate reasons because we could not identify logical groupings and did not want to make assumptions about which reasons should be grouped together [19]. The predominance of reasons reported such as grew apart, arguments, and lack of respect/appreciation suggest a deterioration in the quality of relationships and echoes research over recent decades reflecting the high expectations of self-fulfilment in contemporary marriage and cohabitation and the increasing unacceptability of emotionally and personally unsatisfying partnerships [9, 19, 20, 29].

The finding that men were less likely than women to cite the reason unfaithfulness/adultery (18% *vs*. 24%, respectively) is similar to, but less prevalent than in Natsal-2 (32% *vs*. 41%) [19]. However, changes in reasons between Natsal-2 and Natsal-3 should be interpreted with caution as they refer to partnerships that ended in different time spans (ever *vs*. past 5 years) and different age groups (16–44 years *vs*. 16–74 years) [19]. Finally, as the Natsal studies did not collect data on which partner’s unfaithfulness led to the break-up, the estimates may reflect gender differences in reporting.

One in 20 men and one in 16 women cited the reason sexual difficulties. Natsal-3 data show that sexual function problems are common, and that among individuals in a sexual relationship for the past year, one in five men and women report an imbalance in level of sex interest between partners, and one in six says that their partner has sexual difficulties [30]. This suggests that sexual difficulties may exist while not always being viewed as a primary reason for partnership breakdown. Our estimates of the prevalence of reporting this reason are lower than in Natsal-2 (men 9% and women 12%, respectively), and considerably lower than in the Dutch national survey (41% and 44%) [9], and a recent US study (27% and 22%) [29]. This may be due to measurement differences as participants in the Dutch survey tended to report more reasons (averages of 6.6 *vs*. 1.8 reasons in Natsal-3), while the US study used the same questionnaire as the Dutch and was conducted in a non-representative sample.

The greatest gender difference was found in the proportion citing domestic violence as a reason for the breakdown of their relationship and, in this respect, our research is consistent with those of others in that women are more likely than men to give this as reason [8, 9, 11, 19, 20]. Given our estimate of one in six women reporting domestic violence as a reason for the breakdown is likely to grossly underestimate the role of domestic violence in relationship dissolution, then these data support calls for a greater emphasis on tackling violent partnerships in public health policy and interventions.

### Implications for policy and practice

Accepting that data of these kind can only ever be what people report, then the predominance of reported reasons concerned with communication and a deterioration of the relationship quality suggest that there is a place for promoting better communication and conflict resolution skills in relationship counselling and education [29, 31], including in the context of young people’s sex and relationship education. This recommendation tallies with other data from Natsal-3 which showed that young people desire more information on communication within relationships, and not just the physical aspects of sex [32].

### Unanswered questions and future research

There is a need for qualitative research and longitudinal studies to assess how partnership characteristics and life course events preceding break-up correspond to the reasons reported [9, 20]. Future studies might attempt, where possible, to interview both partners to explore more fully initiation of the break-up, the attribution and weighting of cited reasons. The case can be made for distinguishing between the different types of cohabitations, and also addressing new topics, such as disagreement on the use of social media within the partnership [33].

### Conclusion

In conclusion, our findings show more similarities than differences between recently ended marriages and cohabitations among men and women in Britain. For both types of partnership types, cited reasons relating to communication and relationship quality dominated, followed by unfaithfulness/adultery which, given the data are representative of the general population, support a focus on these topics in the context of changing partnership formation, and socio-cultural shifts in expectations of, and pressures on modern relationships, in preventive and therapeutic interventions addressing live-in partnership breakdown.
